# Behavioural intervention to promote the uptake of planned care in urgent dental care attenders: a feasibility randomised controlled trial

**DOI:** 10.1186/s12903-024-03942-2

**Published:** 2024-02-06

**Authors:** Rebecca Harris, V. Lowers, A. Best, G. Burnside, JE. Clarkson, C. Hulme

**Affiliations:** 1 Department of Public Health, Policy and Systems, Institute of Population Health, University of Liverpool, Whelan Building, Liverpool, L69 3GL UK; 2 Liverpool Clinical Trials Centre, Clinical Directorate, University of Liverpool, Liverpool, UK; 3 Department of Health Data Science, Institute of Population Health, University of Liverpool, Liverpool, UK; 4 Division of Oral Health Sciences, School of Dentistry, University of Dundee, Dundee, UK; 5 Health Economics Group, Department of Health & Community Science, University of Exeter Medical School, Exeter, UK

**Keywords:** Inequalities, Dental practice, Behaviour change, Dental attendance, Primary care, Urgent care

## Abstract

**Background:**

Urgent dental care may be the only place where many people, especially vulnerable groups, access care. This presents an opportunity for delivery of a behavioural intervention promoting planned dental visiting, which may help address one of the factors contributing to a socio-economic gradient in oral health. Although we know that cueing events such as having a cancer diagnosis may create a ‘teachable moment’ stimulating positive changes in health behaviour, we do not know whether delivering an opportunistic intervention in urgent dental care is feasible and acceptable to patients.

**Methods:**

The feasibility study aimed to recruit 60 patients in a Dental Hospital and dental practices delivering urgent care within and outside working hours. Follow-up was by telephone, e mail and post over 4 months.

**Results:**

Although the recruitment window was shortened because of COVID-19, of 47 patients assessed for eligibility, 28 were enrolled (70.1% of screened patients provided consent). A relatively high proportion were from disadvantaged backgrounds (46.4%, 13/28 receiving State benefits). Retention was 82.1% (23/28), which was also the rate of completion of the Oral Health Impact Profile co-primary outcome. The other primary outcome involved linking participant details at recruitment, with centrally-held data on services provided, with 84.6% (22/26) records partly or fully successfully matched. All intervention participants received at least some of the intervention, although we identified aspects of dental nurse training which would improve intervention fidelity.

**Conclusions:**

Despite recruitment being impacted by the pandemic, when the majority of clinical trials experienced reduced rates of recruitment, we found a high recruitment and consenting rate, even though patients were approached opportunistically to be enrolled in the trial and potentially receive an intervention. Retention rates were also high even though a relatively high proportion had a low socio-economic background. Therefore, even though patients may be in pain, and had not anticipated involvement before their urgent care visit, the study indicated that this was a feasible and acceptable setting in which to position an opportunistic intervention. This has the potential to harness the potential of the ‘teachable moment’ in people’s lives, and provide support to help address health inequalities.

**Trial registration:**

ISRCTN 10,853,330 07/10/2019.

## Background

Socio-economic inequalities are an important policy concern because of the principle of distributive justice which is concerned about treating equals equally and giving all people their due [1]. Unfortunately, we find a pattern that is repeated globally: as socio-economic advantage decreases, so does people’s use of dental services, as does their oral health [2]. While the relative contribution of lifestyle and service-related factors to inequalities in oral health can be debated, there is evidence that low social position is tied more strongly to measures of disease management (missing and untreated teeth) than to indicators of oral disease per se, leading to the conclusion that the use and nature of dental services received is key to how low social position translates into inequalities in oral health [3].

There are multi-faceted reasons for the social patterning of dental attendance which involve macro, meso and individual level factors operating together to create a dynamic ‘spider-less web of multiple intersections’ [4, 5]. Examples of macro-level factors are governmental pricing policies for dental care including whether there is free or subsidised care for low-income groups, as well as general employment and welfare policies; whereas meso-level factors include factors such as the extent of close neighbourhood social ties, how many dental services there are in the local area, and what the transport system is like [4]. These interact with individual level factors such as self-efficacy (how able some-one feels that they can use dental services), dental anxiety, and a range of other psychological factors [4]. However, this presents us with a bewildering range of possible intervention points. The resultant complexity of the area may explain why reports of interventions addressing inequalities in the use of planned dental care are so few, and at odds with the global nature of the problem [6]. Krieger’s advice, however, is to start by tackling factors most amenable to ‘practical intervention’ and nearest to outcomes of the pathways involved [5]. For dental visiting, this means that a promising place to start would be positioning an intervention where people have turned to urgent dental care because of problems, and addressing some of the socio-psychological factors which shape individuals’ motivation and intentions to do with taking up planned care. This means that the intervention involved did not address provider or service-related reasons why individuals do not attend for planned care, which were out with its scope, while still important issues in themselves.

‘Teachable moments’ are “cueing events”: i.e. naturally occurring health events or circumstances that lead individuals to make health behaviour changes [7]. The experience of dental problems forcing the individual to visit a dental service may act in the same way as cueing events such as a cancer diagnosis on smoking cessation behaviour [8], although we do not know whether both the setting or people’s experience of pain or other problems at the time of intervention delivery means that this is not the right moment for other reasons. A behavioural intervention to be delivered in urgent dental care settings was developed in conjunction with extensive public and patient involvement [6], and so this feasibility study was undertaken to address key areas of uncertainty before a full randomised controlled trial was undertaken.

The feasibility trial had several primary objectives. Firstly, to investigate the likely rate of completeness of co-primary outcome data: attendance at an NHS dental practice as measured by routine data collected by NHS Business Services Authority (objective 1); and completeness of participant self-reports of oral health quality of life (objective 2). Also, to identify if recruitment of patients to the study and opportunistic delivery of the intervention when attending for urgent dental care would yield a reasonable recruitment rate (objective 3); and to explore the fidelity of the intervention as delivered by trained dental nurses in the urgent dental care setting (objective 4).

## Methods

Favourable ethical approval was granted in November 2019 by the London-Bromley Research Ethics Committee (19/LO/1510).

### Trial design

The study was a randomised, two-arm, parallel study. Allocation to intervention (the RETURN behavioural intervention aimed at supporting planned dental visiting) and control (usual care per site) was 1:1. This was an open study since investigators involved in intervention delivery and participants were not blind to allocations. Participants were followed up for 4 months after randomisation.

### Participants

#### Inclusion criteria

1. Adults (aged 18 years or over) attending for urgent dental care.
2. Has not visited an NHS or private dentist for a non-emergency appointment (i.e. when not in pain or symptomatic) for 2-years or more.
3. Willing to spend time completing assessments and receiving the intervention.
4. Able to provide a telephone number, e mail or postal address to allow follow-up.
5. Has provided written informed consent.

#### Exclusion criteria

1. Do not adequately understand spoken and written English.
2. Not responsible for making own dental appointments: i.e. done by a carer.
3. Attends for planned dental care appointments i.e. confirms they have attended an appointment when not in pain or with symptoms within the past 2-years.
4. Have previously been enrolled in the RETURN programme.
5. Recruited to another clinical dental trial only if considered not to have any detrimental effect on the RETURN trial.
6. Lives with or is related to somebody participating in the RETURN study.

### Study settings

Recruitment was in Cheshire and Merseyside in North-West of England in sites providing urgent dental care: (i) a secondary care Teaching Dental Hospital setting (Liverpool University Hospitals Foundation Trust, LUHFT); (ii) in a primary care service contracted to provide urgent dental care out of hours (OH); and (iii) in an NHS dental practice contracted to provide urgent care within working hours (IH). There were no recruitment targets per site. At the time of the feasibility study there was good availability of services providing planned care appointments for new National Health Service (publicly funded) patients.

### Participant identification, screening, and consenting

Patients attending for urgent care who did not have their own dentist were classified as potentially eligible. They were approached on arrival by trained dental team (DT) members (dental nurses or other staff employed at the site) to screen for inclusion in the trial and prior to seeking consent, using questions routinely asked during usual care such as whether the patient had their own dentist and how long it had been since they had visited a dentist for a non-urgent appointment. Consent was then sought by trained staff (DT or research nurses) using a patient information sheet outlining risks and benefits of being involved in the study, and then a final study eligibility check was undertaken. Reasons for non-eligibility and non-consent were gathered using a paper-based screening log.

### Intervention

The RETURN intervention is described in detail elsewhere [6, 9]. Participants were shown a pack of resources which included 6 booklets outlining different barriers or reasons people often gave for not taking up planned dental care (‘I don’t have time’; ‘I don’t think to go when I’m not in pain; ‘I don’t have trust in dentists’; cost; embarrassment; anxiety). These 6 identified barriers were the result of a prior literature review and synthesis of theory as well as qualitative and public and patient engagement work testing how barriers to planned dental care were experienced within the context in which the intervention was to be applied (adults living in the city of Liverpool, England and the surrounding areas who attended urgent services for their dental care), [6]. In a non-judgemental and listening conversation with trained dental nurses, participants identified which of these main reasons were why they did not make planned dental visits and watched an accompanying video clip of a few minutes where someone spoke about their experience about that barrier. Dental nurses then supported participants in setting a planned dental visiting goal and accompanying action plan. While participants might have felt more than one of the barriers were relevant to them, because of time, the discussion was focused on one main barrier identified, although participants were able to access material on other barriers with the whole pack given to them to take home after that visit. Participants also received a text a few days later which reminded them of the action plan they had set.

### Outcomes

A feasibility study asks whether the study can be done, whether it should proceed, and if so, whether there needs to be any changes before a bigger study measuring intervention effect is undertaken. Clear progression, or stop/go criteria are usually set a priori at the feasibility protocol stage which inform the decision as to whether a definitive study should follow [10]. Progression criteria often take the form of traffic light ratings, where green (go) indicates that the criteria have been met and the trial should proceed, amber (amend) indicates that amendments should be made to the design before embarking on a larger trial, and red (stop) indicates that there needs to be careful consideration as to whether a main trial is advisable [10]. Outcomes with pre-specified stop/go criteria used to judge whether to proceed with a definitive trial are summarised in Table 1.

#### Proposed primary outcome measures for the full trial (objectives 1 and 2)

For the main trial co-primary outcomes had been identified: the first (attendance at a dental practice for a planned care appointment within 12 months) involved data routinely collected by the NHS Business Services Authority (NHSBSA) which is an organisation responsible for collecting data on the activity undertaken by dentists with contracts to deliver dental care in NHS dental practice. Since data entered onto the NHSBSA system is linked to dentists’ payment, then a relatively complete dataset was expected, although this would be subject to a successful data linkage between participant details collected at recruitment with appropriate identifiers in NHSBSA data. Although follow-up in the main trial was planned for up to 18 months after recruitment, since the feasibility trial had a follow up period of 4 months, the feasibility study investigated the proportion of participants recruited who had been matched with records of the urgent care (baseline) visit. Since NHSBSA only holds records for NHS dental practice, participants recruited in LUHFT were excluded from those with expected matches. NHSBSA data also does not collect data where primary dental care is provided privately.

The second co-primary outcome involved self-reported oral health quality of life as measured by the 14-item Oral Health Impact Profile (completeness taken as 12 or more questions answered), [11], and allowed outcome data to be collected from participants attending private dental services and secondary care, although this would be subject to non-response bias if follow-up attrition rates were high. Oral Health Impact Profile (OHIP) data was collected for all participants as part of baseline assessments, and in follow-up data collection which was undertaken firstly by telephone, and where a phone number was not supplied, via email. If an e mail address was not provided, or where there was no response by other means, a last resort of postal contact was used.

#### Recruitment rate (objective 3)

Number of participants recruited across all types of sites with breakdown given by type of site.

#### Fidelity (objective 4)

Observations by a member of the research team of intervention delivery to trial participants by a trained DT member to determine percentage allocated participants receiving at least some of the intervention.

**Table 1:** Trial objectives, outcome measures, pre-specified criteria as to whether to proceed to main trial

Objectives	Outcome Measures	Time point(s) of evaluation	Stop / Go Criteria	Stop / Go Categorisation
1. To identify rate of matching patient ID in BSA routine records and likely missing outcome data	Matching patient name, date of birth, gender and contract number the service is delivered under against record of attending for NHS urgent dental practice care in the Business Service Authority (BSA) database The denominator is those recruited at baseline from NHS dental practice who would be expected to be in the BSA system	Baseline and 4 months (where possible)	≥ 95% successfully matched	Green
			90–95% successfully matched	Amber
			< 90% successfully matched	Red
4. To identify rate of completeness of valid OHIP outcome data	Percentage of participants providing valid* OHIP data at baseline and follow up (i.e. respond to 12 items or more)	Baseline and 4 months	≥ 80% of participants	Green
			60–80% of participants	Amber
			< 60% of participants	Red
7. To identify if recruitment rates are feasible	Number of patients recruited across all types of sites	Baseline	60 patients recruited	Green
			40–59 patients recruited	Amber
			< 40 patients recruited	Red
10. To determine fidelity of intervention delivery	Observations of participants to determine % allocated patients who are observed to receive at least some of the intervention (receive at least some of the intervention material)	Baseline	≥ 80% allocated patients who are observed receive at least some of the intervention material (either/and booklet or online)	Green
			60–80% receive at least some of the intervention material	Amber
			< 60% receive at least some of the intervention material it	Red

*Valid: 12 or more questions answered.

*Stop / Go criteria key: Green*: No action required; *Amber*: Consider strategies to improve outcome; *Red*: Consider whether full study is feasible

### Health economics

A common key objective in any full randomised controlled trial is an economic evaluation. In the case of the RETURN intervention trial, the anticipated an economic evaluation was to be limited to the patient rather than a societal perspective and be based on quality adjusted life years (QALYs) derived from the EQ-5D-5 L questionnaire. The economic analysis would also require completion of an assessment of costs gathered by a health economics patient questionnaire, with data fields ranging from the number of healthcare contacts within the last eight weeks for teeth / mouth problems (at a variety of services), medications (prescription and non-prescription), travel expenses associated with teeth/mouth problems, days lost from work, earnings lost, payments and other expenses related to teeth/mouth problems. The feasibility study included collection of health economics data to assess whether participant burden was acceptable, although this did not feature as a primary outcome of the feasibility trial with progression criteria. Patient burden was assessed by noting the completeness of data at baseline and also by asking participants about burden during follow-up telephone calls.

### Sample size

As this was a feasibility study, a formal sample size calculation was not required; because a sample size calculation suggested we needed to recruit 1180 participants for the main trial, the sample size for the feasibility study was set at 60 participants (30 randomised to Intervention and 30 to Control), [12]. Assuming the observed proportions falls within our “green” range stop/go criteria for collection for co-primary outcomes, a sample size of 60 would ensure that 95% confidence intervals are within +/- 10%. No interim analyses or stopping guidelines were planned.

### Randomisation and blinding

Participants were equally randomised to the intervention or control group in a 1:1 ratio using a secure (24-hour) web-based randomisation programme controlled centrally. Randomisation lists were generated using block randomisation with random variable block length, stratified by site. The lists were produced by an independent statistician (who was not otherwise involved in the RETURN trial). Due to the nature of the intervention, staff at sites, and researchers carrying out follow-up were not blinded to allocation.

### Analytical methods

Statistical analysis was focused on assessing the stop/go criteria for progression to the full trial (Table 1), including descriptive statistics for each of the outcomes, assessed against the pre-specified stop/go criteria. No statistical inference was carried out for this feasibility study.

## Results

### Recruitment

Recruitment started in the IH site on 27.1.20; in the OH site on 16.2.20 and in LUHFT on 12.3.20. In the IH site, the last participant randomised was on 26.2.20 after 9 recruitment sessions and 20 participants were recruited. In the OH site, after 3 recruitment sessions and 6 participants recruited, recruitment finished on 22.2.20. In LUHFT recruitment was over 2 sessions (2 participants recruited) and was halted on 16.3.20. The recruitment period finished prematurely in the OH site and LUHFT because of COVID restrictions to face-to-face research conducted in healthcare settings.

### Participant flow

**Fig. 1:**
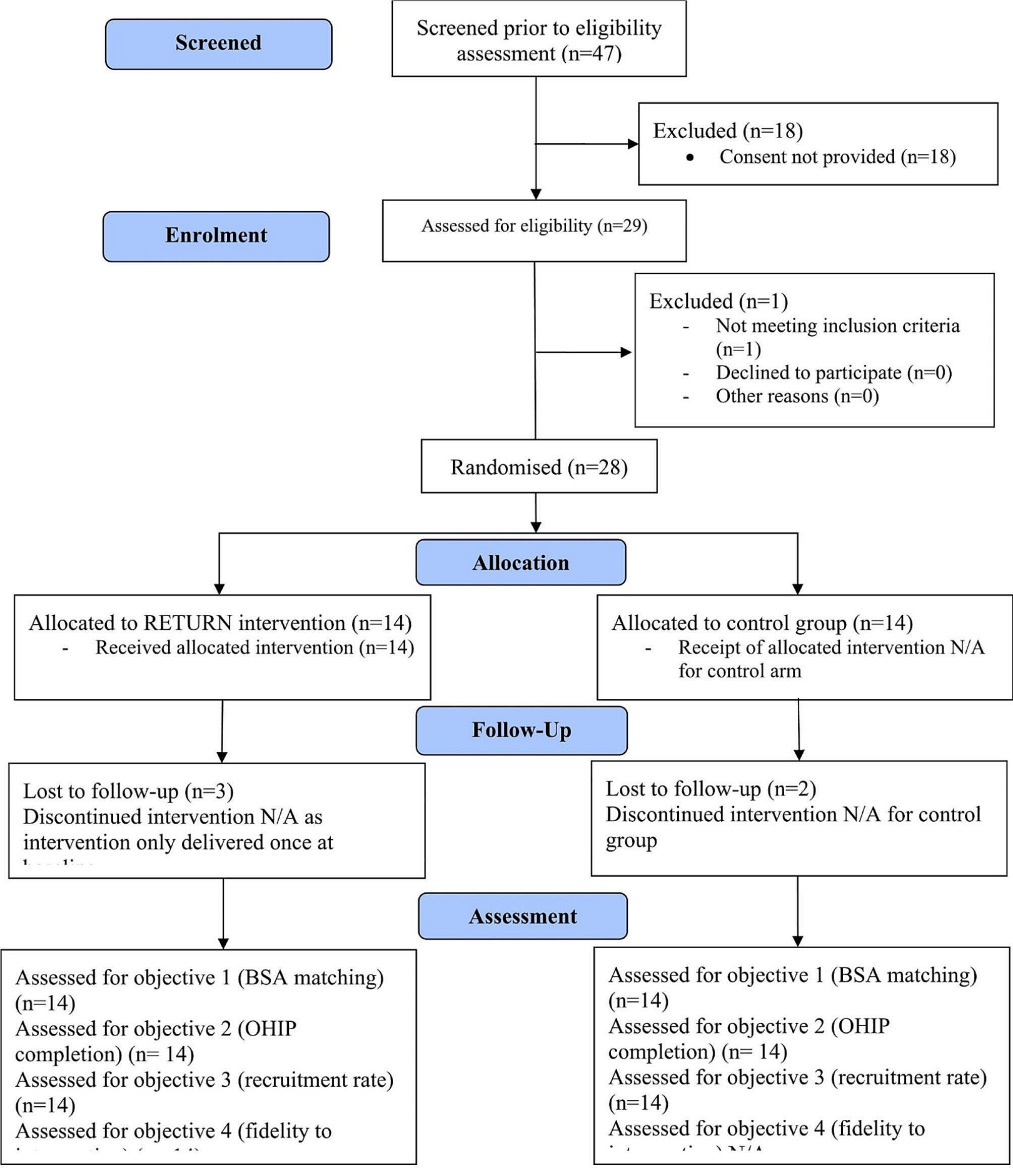
Participant flow diagram

Forty-seven patients were approached and assessed for eligibility, with 61.7% (29) identified as potentially eligible for inclusion in the trial on screening. One further patient was found not to meet inclusion criteria after consenting and before randomisation (Fig. 1). Six patients approached who did not provide consent was because of eligibility reasons (2 were aged < 18 years, 3 had insufficient ability to understand written/ spoken English, and 1 said they had visited an NHS or private dentist when not in pain in the last 2 years). Seven patients did not consent because they did not want to take part in research; 2 said they were unable to concentrate at that time because of their pain, and 3 said they did not have enough time that day. If ineligible patients are excluded, consenting rate was 70.1% (29/41).

### Loss to follow-up

Twenty-three (82.1%) participants were successfully followed up at 4-months after randomisation; 11 of which were in the Intervention arm and 12 in the Control.

### Baseline data

Table 2 shows that participants were evenly distributed by gender (50%, 14 male; 50%, 14 female) and had a median age of 34.9 years (IQR 25.4–43.5 years). The majority 78.6% (22) were White British. Almost half (46.4%, 13) were in receipt of state benefits, with only 12 out of the 28 participants reporting they were employed. Over half (53.5%, 15) only had qualifications usually obtained at school up until the age of 16 years.

**Table 2:** Baseline data: Demographic and clinical characteristics for each group

Demographic	Baseline characteristics	RETURN Intervention	Standard Care	Overall
Gender, n (%)	N	14	14	28
	Female	8 (57.1%)	6 (42.9%)	14 (50.0%)
	Male	6 (42.9%)	8 (57.1%)	14 (50.0%)
Age (years) Summary	n (n missing)	14 (0)	14 (0)	28 (0)
	Mean (S.D.)	33.1 (9.9)	35.7 (11.6)	34.4 (10.7)
	Median (Q1-Q3)	34.9 (24.9 to 43.0)	33.1 (28.7 to 45.3)	34.9 (25.4 to 43.5)
	Min-Max	18.7 to 46.7	19.2 to 57.5	18.7 to 57.5
Ethnicity, n (%)	N	14	14	28
	Asian or Asian British: Indian	1 (7.1%)	1 (7.1%)	2 (7.1%)
	Asian or Asian British: Pakistani	1 (7.1%)	0 (0.0%)	1 (3.6%)
	Mixed/ multiple ethnic groups: Any other Mixed/Multiple ethnic background	0 (0.0%)	1 (7.1%)	1 (3.6%)
	Mixed/ multiple ethnic groups: White and Black African	0 (0.0%)	1 (7.1%)	1 (3.6%)
	White: Any other white background	1 (7.1%)	0 (0.0%)	1 (3.6%)
	White: English/Welsh/Scottish/Northern Irish/British	11 (78.6%)	11 (78.6%)	22 (78.6%)
Highest qualification, n(%)	N	14	14	28
	No formal qualifications	2 (14.3%)	4 (28.6%)	6 (21.4%)
	GCSEs / O Levels (any grade), NVQ Level 1 or similar	5 (35.7%)	4 (28.6%)	9 (32.1%)
	2 + A Levels/NVQ Level 3 or similar	4 (28.6%)	3 (21.4%)	7 (25.0%)
	Undergraduate degree (BA, BSc etc.)	0 (0.0%)	1 (7.1%)	1 (3.6%)
	Postgraduate degree or similar (PGCE, MA, PhD etc.)	3 (21.4%)	2 (14.3%)	5 (17.9%)
Employment Status, n(%)	N	14	14	28
	Employed or self employed full-time (30 h or more/week)	6 (42.9%)	2 (14.3%)	8 (28.6%)
	Employed or self employed part - time (Less than 30 h/week)	2 (14.3%)	2 (14.3%)	4 (14.3%)
	Reason for not working provided	6 (42.9%)	9 (64.3%)	15 (53.6%)
	Data unobtainable	0 (0.0%)	1 (7.1%)	1 (3.6%)
Reasons for not working, n(%)^1^	N	6	9	15
	Currently looking for paid work	2 (33.3%)	1 (11.1%)	3 (20.0%)
	Not currently looking for paid work (e.g. Looking after family)	0 (0.0%)	1 (11.1%)	1 (6.7%)
	Student	3 (50.0%)	2 (22.2%)	5 (33.3%)
	Unable to work Due to disability	1 (16.7%)	4 (44.4%)	5 (33.3%)
	Other^2^	0 (0.0%)	1 (11.1%)	1 (6.7%)
Receipt of benefits, n(%)	N	14	14	28
	No	10 (71.4%)	4 (28.6%)	14 (50.0%)
	Yes	4 (28.6%)	9 (64.3%)	13 (46.4%)
	Data unobtainable	0 (0.0%)	1 (7.1%)	1 (3.6%)

^1^ Denominator for percentage is those that have provided reason for not working

^2^ Other reason provided: unemployed

### Outcomes

#### Objective 1: rate of matching of participant data at baseline visit to centrally held NHS BSA data

Since 2 participants were recruited at LUHFT, with no matching records in the BSA system expected the number of participants included in this analysis was 26. Based on participant name, gender and date of birth, 22 matching participant records were retrieved. One further record did not have a treatment completion date matching the date of the baseline appointment, although a matched record was found which fell before randomisation and still during the study period. A matching rate of 84.6% (22/26) for fully or partially matched records, or 80.8% (21/26) fully matched was found. The following 4 records were excluded as successful matches: one which was linked to multiple records based on the participants’ identifier; and 3 records matching BSA identifiers but which were coded against non-urgent visits.

Progression criteria for the main trial were set as > 90% successful linking of participant identifiers collected at randomisation (Table 1) to centrally held data about visits to NHS dentists. This was based on national testing of the accuracy of the NHSBSA unique patient identifier system which reported an inaccuracy of unique patient identifiers to be < 1% of a practice list (for example, because of misspelling the patient’s name) [13]. Discussion with NHSBSA identified that the accuracy of matching would be improved in the main trial by using an additional identifier of postcode, and by checking the spelling of names and checking of dates of birth at each follow-up point.

#### Objective 2: rate of completeness of OHIP outcome data

OHIP data was missing for one participant (in the Control arm) at baseline, and for 5 participants at follow-up (3 intervention, 2 control), Table 3. This gave a rate of completeness of 82.1% (23/28) which met trial progression criteria (Table 1).

**Table 3:** Oral Health Impact Profile (OHIP) data completed at baseline and follow-up

Details	Summary	RETURN Intervention	Standard Care	Overall
Baseline OHIP-14 score Summary	n (n missing)	14 (0)	13 (1)	27 (1)
	Mean (S.D.)	23.3 (15.8)	22.7 (13.3)	23.0 (14.4)
	Median (Q1-Q3)	23.5 (10.0 to 35.2)	17.2 (14.0 to 30.0)	18.0 (11.0 to 35.2)
	Min-Max	0.0 to 50.0	5.0 to 46.0	0.0 to 50.0
Follow-up OHIP-14 score Summary	n (n missing)	11 (3)	12 (2)	23 (5)
	Mean (S.D.)	15.5 (13.3)	18.8 (14.0)	17.2 (13.4)
	Median (Q1-Q3)	10.0 (8.0 to 22.0)	19.5 (5.5 to 30.5)	13.0 (8.0 to 27.0)
	Min-Max	0.0 to 47.0	2.0 to 43.0	0.0 to 47.0
Change in OHIP-14 scores Summary (follow up - baseline)	n (n missing)	11 (3)	11 (3)	22 (6)
	Mean (S.D.)	-7.7 (17.2)	-4.5 (12.2)	-6.1 (14.6)
	Median (Q1-Q3)	-1.0 (-16.0 to 4.0)	-3.0 (-7.0 to 4.0)	-3.0 (-11.0 to 4.0)
	Min-Max	-42.0 to 12.0	-35.0 to 9.8	-42.0 to 12.0

#### Objective 3: to identify whether recruitment rates are feasible

Twenty-eight participants were recruited 47% of the way through the 4-month recruitment window, and despite a staggered opening of sites which meant that the OH site was only open for a 6-day period and the LUHFT site for 4 days. Although recruitment finished prematurely because of COVID restrictions so that the feasibility target was not reached, recruitment to that point had already demonstrated that recruitment rate in urgent dental care was in line with trial progression criteria.

#### Objective 4: to determine fidelity of intervention delivery

The number of participants recorded (by the dental nurse (DN) delivering the intervention) to have engaged with at least some of the intervention was 100% (14) of intervention patients. Eleven of the 14 intervention delivery sessions were observed by a researcher (VL) who assessed fidelity according to different domains:

i) Adherence (whether intervention components were delivered to patients): All 11 (100%) participants received the booklet pack and a text message one week after recruitment. Ten (91%) selected and went through at least one barrier booklet with a nurse, selected and watched a barrier video and set a goal and made an action plan. One participant received a lot of urgent treatment just prior to intervention delivery and was in pain, so was only given the pack to take away on the day of recruitment. Only 5 (45%) participants observed however, had a listening, non-judgemental conversation with the nurse exploring how the patient felt about their identified barrier, which meant that further training was needed related to this aspect of intervention delivery before the main trial.
ii) Exposure (how ‘much’ of the intervention patients received) and quality / competence of the intervention delivery: Observations in two types of sites showed slight variation with intervention delivery in IH taking a median of 20 min (interquartile range [IQR] 15.5–30.0 min) or between 13 and 37 min; and OH taking a median of 11 min (IQR = 10.5–17.5 min) or between 10 and 24 min. The biggest factor influencing intervention delivery time was the extent the nurses engaged the participants in conversations around patients’ barriers, with the OH recruiting nurse generally using fewer probing questions. Observations also found nurse confidence was a further factor influencing exposure, with the IH recruiting nurse generally less confident in delivery, and not always guiding the participant towards the intervention material most in line with patients’ concerns. Taken together, intervention exposure was probably equivalent between sites, although this identified that some aspects of nurse training requiring strengthening before a main trial.
iii) Patient responsiveness (enthusiasm, comprehension and application of skills): Participant responsiveness to the intervention varied. Where participants’ concerns were closely aligned with the material, engagement and application of skills was highest. This emphasised the importance of the nurse conversation in eliciting patients’ concerns appropriately and helping orientate them towards the most meaningful material:
  P: “I know I need to go to the dentist, but I will never be able to find a dentist that I can trust.
  DN01: “We do have a booklet that’s all about trust”
  P “I’m so surprised that there is a booklet called Trust and that other people feel this way.”
*Observation: The patient appeared very interested in the booklet and was looking at the front cover. At the end of intervention delivery session, the patient said she was amazed that others felt like her and this was a comfort. She said she would definitely try to find a dentist.*
  Observation 07, Dental Practice 02.

#### Health economics

Participant burden was a potential concern because of the setting and timing of the study in urgent dental care, and because a relatively high proportion of participants were anticipated to be from low educational backgrounds. Participant burden was explored in participants who completed follow-up by telephone. All participants contacted reported that they were happy with what was asked of them during their participation in the trial. When asked about the length of time the trial took at their urgent care appointment, just one intervention patient stated that it felt long, but acknowledged that this is not time they would have spent by themselves and so it was useful. One control patient mentioned that it felt long because they were in pain, but that they were still happy to take part. No patients reported the follow-up too burdensome, and all said they were happy to have taken part. When asked specifically about the questionnaires, all patients reported they were happy with the questions that had been asked of them, and that there were no questions that had felt inappropriate or that they did not want to answer.

## Discussion

Emergency departments (EDs) or services which provide urgent dental care are an important part of health care systems - being in many respects, the “safety net” of the system, and the place where several types of vulnerable groups come to access care [14]. Unfortunately, their use is on the rise, especially among certain population groups such as 18-44-year olds and those with low income [15]. Opportunistic interventions, such as brief interventions for alcohol dependent patients attending EDs, have been shown to be timely and effective [16]. For example, Chafetz et al. found that after initiating contact with the alcohol-dependent patients attending EDs, 65% of patients randomised to the treatment group made a follow-up visit to the alcohol clinic, compared with 5% in the control group [17].

Although an opportunistic intervention for patients seeking urgent care for dental problems had not been previously explored, we found that a relatively high proportion (70% of eligible patients), consented to participate in the trial. This was despite a high proportion being from a low income (46% on state benefits) and low educational (53% left school at 16 years) background, and other literature showing that participation in clinical trials tends to be disproportionately low in such groups [18]. Minority ethnic groups are also often found to be less likely to participate in clinical trials [18]. Although 78.6% of trial participants had a White British ethnicity, this proportion was actually *lower* than the national census statistics for the proportion of White British people living in the Liverpool area (84%), [19]. This is despite feasibility inclusion criteria limiting participation to sufficient English language ability in order to understand materials and take part, which was introduced for cost and pragmatic reasons.

The feasibility study took place as pandemic storm clouds were gathering, which led to an early closure of the recruitment window, meaning that the full recruitment target was not reached. Recruitment rate was potentially impacted by COVID-19 related staff sickness and isolation, and diversion of staff resources and focus on the study at sites. Staff reported that numbers of people attending for urgent dental care had started to decline in late February/early March 2020 because of population efforts to limit anything other than essential travel and use of services. This appears to have been an impact of the pandemic seen elsewhere, with attendances at emergency dental services in Beijing, China reduced by 38% at the beginning of the pandemic [20]. Moreover, there was pandemic disruption to non-COVID-related clinical trials generally. For example, less than 20% of Oncology trials in the United States and Europe continued to enrol patients at the same rate; partly because of the necessity to divert clinical staff to patient care, and the focus on COVID-related trials, and partly because of operational challenges related to limiting in-person visits to minimize potential viral exposure [21]. That we managed to achieve such a high accrual rate in such circumstances is indicative of the ability to achieve high recruitment rates in a full RCT under more usual circumstances.

Phase II studies are primarily designed to test the feasibility and acceptability of various methods before a larger definitive phase III RCT is undertaken. One aspect we explored was the feasibility of using routinely held NHSBSA data for a primary outcome, and the reliability of linking personal identifiers collected in the urgent dental care setting. Relatively few trials have made use of these data in England [22], and although we experienced a relatively high matching rate, we identified that by adding postcode as an additional identifier, this could be further improved.

Dental nurses are key members of dental practice teams, and potentially positioned to be the most appropriate, effective and cost-efficient member of staff to deliver behaviour change conversations to patients. Previous trials involving DNs being trained to deliver theoretically informed behaviour change conversations have mostly involved young children as the participants concerned, with findings promising in terms of outcomes [23, 24]. Nevertheless, other studies report that dental teams find behaviour change conversations difficult to initiate and are uncertain about acquiring sufficient skill through brief training [25]. While clinical guidelines identify that dental professionals have a role supporting patients to build their motivation to optimise oral health behaviours, and a range of training resources are available to facilitate this [26], findings from our study indicate that in order for DNs to achieve the required level of competence and confidence to deliver behaviour change conversations effectively, the training required, including some personalised support and shadowing in dental practice, may be considerable – at least for some nurses. Thus, a research question arises, as to the cost-effectiveness and clinical effectiveness of changing dental professionals’ skills in supporting behaviour change as part of clinical practice and, in turn, patient health behaviours and outcomes [25]. The type and intensity of the training required is be a key factor to consider in trials of this type.

Updated UK Medical Research Council guidance confirms the importance of feasibility studies as one of the four main phases in the design and evaluation of complex interventions [27]. Guidance to standardise reporting of feasibility studies is also available [12], which includes a reporting of progression criteria to determine how the results of a feasibility study should be interpreted. Setting these clearly in advance helps the process of identifying potential limitations in a future trial, and what sort of mitigation measures might helpfully strengthen it, although many previous feasibility studies have failed to include progression criteria in their protocols [28]. Where there are several feasibility outcomes with associated progression criteria which form a matrix such as in Table 1, the overall decision as to whether or not to proceed is usually based on the relevant importance of each outcome to overall success (for example, adequate recruitment will be one of the most crucial factors, to ensure resources are not wasted by undertaking an inadequately powered main trial); and whether mitigating measures can be put in place to strengthen any identified weaknesses.

As the MRC complex intervention guidance points out, each phase in the evaluation of complex intervention is heavily context dependent [27]. Questions should be asked at every point concerning how the intervention is interacting with its context, and what key uncertainties remain. Thus, when interpreting feasibility outcomes against progression criteria, there needs to be some discussion regarding context when concluding as to whether progression to a larger trial is advisable. Given that this feasibility study took place in a pandemic context, this is especially important. Attendance rates in urgent dental care, lockdown and staff sickness during the pandemic are all likely to have influenced recruitment rates. On the other hand, while the numbers recruited to the feasibility study (*n* = 28) fell into the red progression criteria category for outcome 3 (n = < 40), this was within half of the recruitment window. This left us, after the feasibility study, with some uncertainty as to the rate of recruitment in a non-pandemic context, which presented an area of potential risk to a successful larger trial.

Internal pilot studies are a mechanism whereby main trials with an element of risk (e.g. where recruitment to time and target is uncertain), can proceed [28]. The main trial goes ahead following a main trial protocol, but progress is assessed early on against pre-specified targets, and there is an agreement as to in what circumstances the study should be stopped, if further information indicates that the main trial is unlikely to reach its recruitment, retention or site set-up targets [28]. Unlike an external pilot, data collected during the internal pilot contribute towards the final trial. So, while this feasibility study suggested that progression of the RETURN intervention to a main trial was advisable, the use of an internal pilot to assess recruitment rates in a non-pandemic context reduces risk. The main trial protocol for the intervention is now published [9] and has progressed with the inclusion of an internal pilot milestone relating to recruitment rate [12]. Exclusion criteria to the main trial includes exclusion of people who had previously participated in the feasibility study reported here.

## Data Availability

The datasets used and analysed during the current study are available from the corresponding author on reasonable request.
